# Supplementary material for: Unperturbed expression bias of imprinted genes in schizophrenia
Source: Nat Commun. 2018 Jul 25;9:2914. doi: 10.1038/s41467-018-04960-9 (PMC6060121; doi:10.1038/s41467-018-04960-9)
Supplement: Supplementary file 1 — Supplementary Information [file 41467_2018_4960_MOESM1_ESM.pdf]

Unperturbed Expression Bias of Imprinted Genes in Schizophrenia (Supplementary Information)

Attila Gulyás-Kovács[1,2,‡], Ifat Keydar[1,2,8,‡],
Eva Xia[1,3], Menachem Fromer[2,4,9], Gabriel Hoffman[2], Douglas Ruderfer[2,4,10],
CommonMind Consortium[§], Ravi Sachidanandam[5],
Andrew Chess[1,2,6,7,*]

Icahn School of Medicine at Mount Sinai (ISMMS)

1 Department of Cell, Developmental and Regenerative Biology, ISMMS

2 Institute for Genomics and Multiscale Biology, Department of Genetics and Genomic Sciences, ISMMS

3 Neuroscience Program, The Graduate School of Biomedical Sciences, ISMMS

4 Division of Psychiatric Genomics, Department of Psychiatry, ISMMS

5 Department of Oncological Sciences, ISMMS

6 Fishberg Department of Neuroscience, ISMMS

7 Friedman Brain Institute, ISMMS

8 Present affiliation: The Simon And Katya Michaeli Bioinformatics Laboratory For The Research Of The Genome Department of Human Molecular Genetics & Biochemistry, Sackler Medical School, Tel Aviv University

9 Present affiliation: Verily Life Sciences

10 Present affiliation: Division of Genetic Medicine, Departments of Medicine, Psychiatry and Biomedical Informatics, Vanderbilt University

‡ equal contribution

§ full list of consortium members appears in the Author Information section of the main text

∗ correspondence: andrew.chess@mssm.edu

1 Supplementary Figures

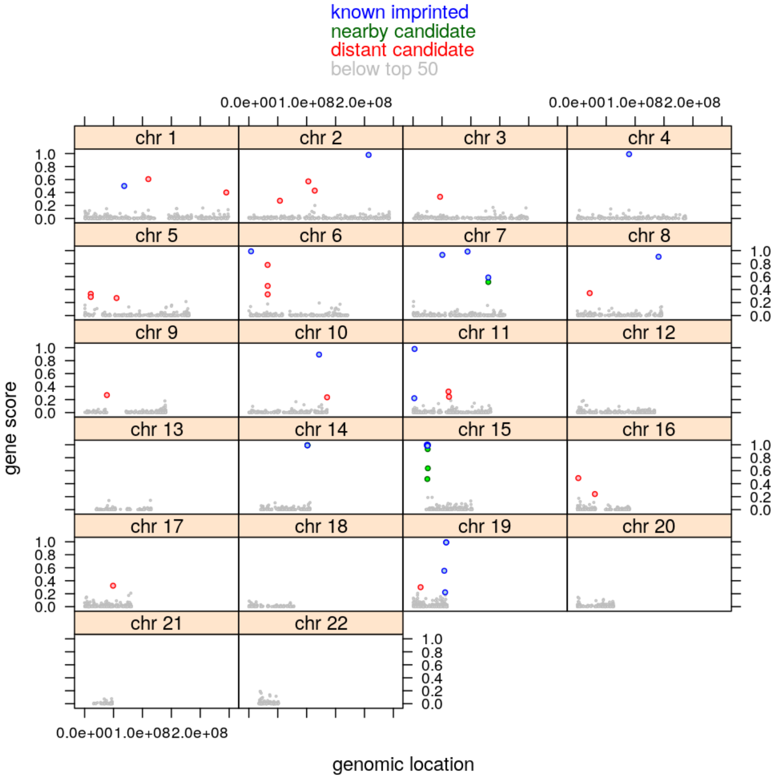

Supplementary Figure 1: Clustering of top-scoring genes in the context of human DLPFC around genomic locations that had been previously described as imprinted gene clusters in other contexts.

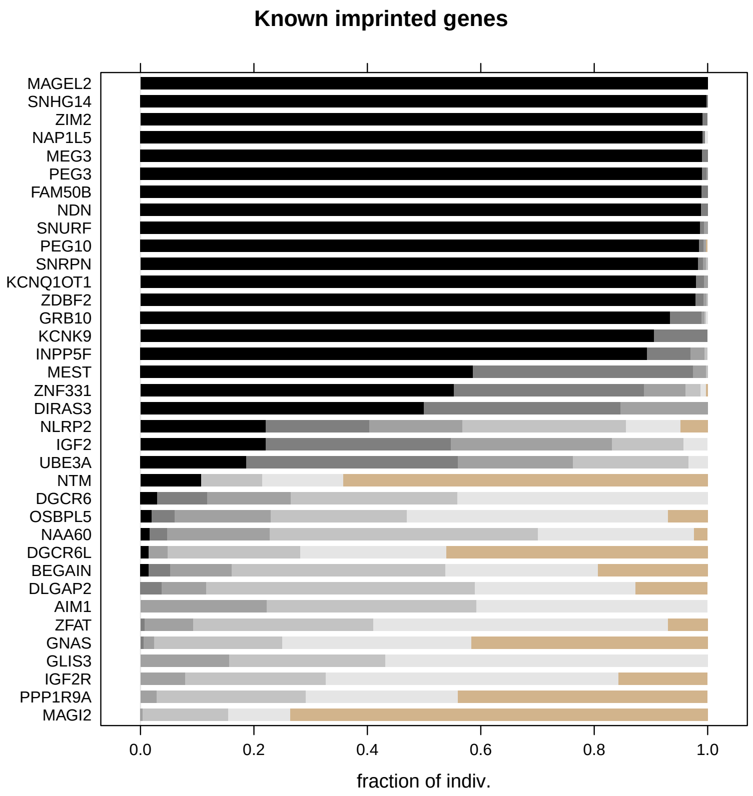

Supplementary Figure 2: Known imprinted genes ranked by the gene score (dark blue bars). "Known imprinted" refers to prior studies on imprinting in the context of any tissue and developmental stage. The length of the black bars indicates the fraction of individuals passing the test of nearly unbiased expression.

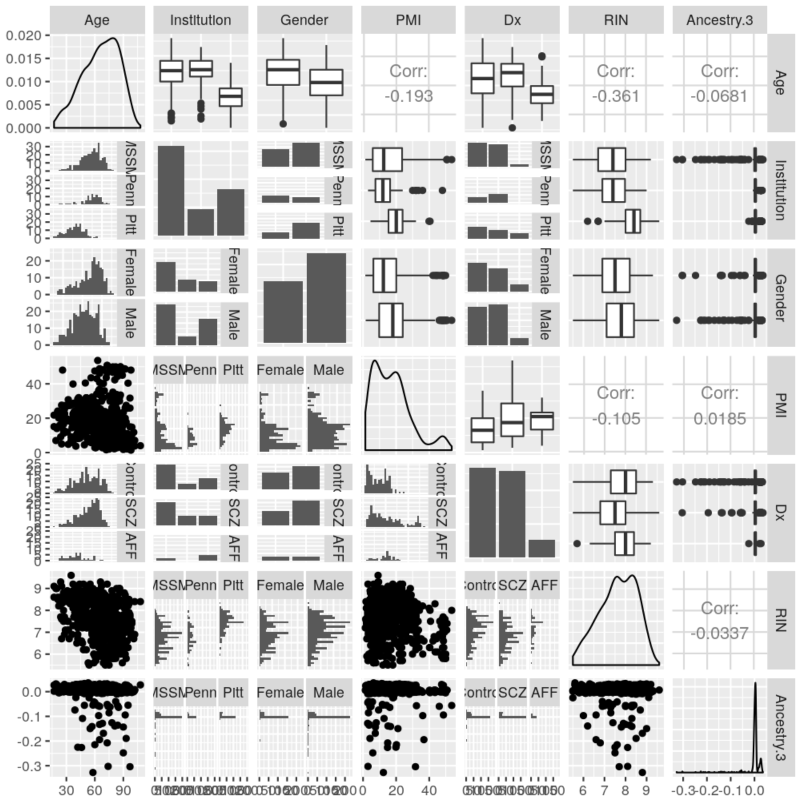

Supplementary Figure 3: Distribution and inter-dependence of explanatory variables. The diagonal graphs of the plot-matrix show the marginal distribution of six variables (Age, Institution,...) while the off-diagonal graphs show pairwise joint distributions. For instance, the upper left graph shows that, in the whole cohort, individuals' Age ranges between ca. 15 and 105 years and most individuals around 75 years; the bottom and right neighbor of this graph both show (albeit in different representation) the joint distribution of Age and Institution, from which can be seen that individuals from Pittsburg tended to be younger than those from the two other institutions.

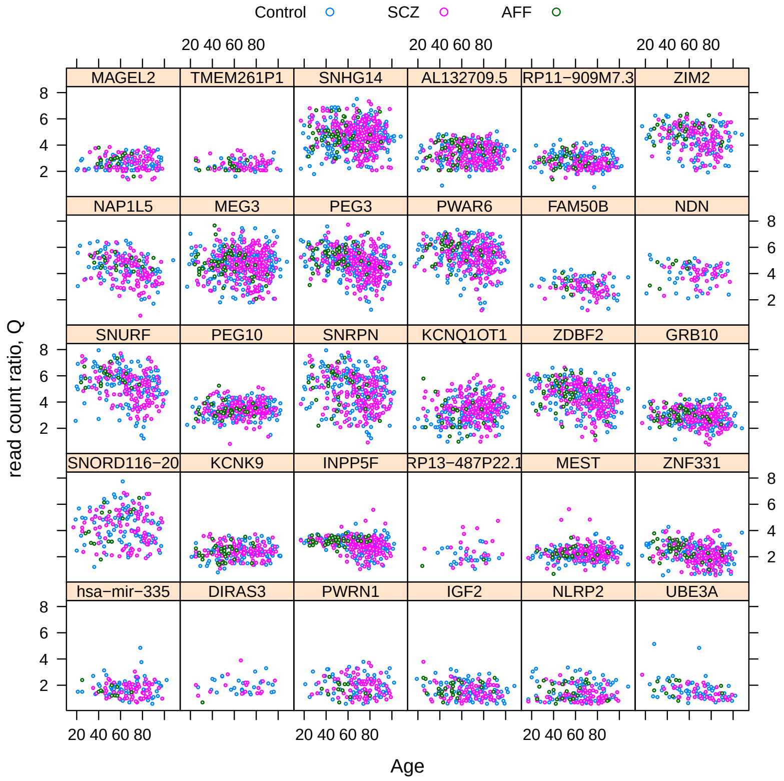

Supplementary Figure 4: The quasi-log transformed read count ratio Q and age for imprinted genes. See Fig. 5 for the corresponding plots without quasi-log transformation and note that statistical inference was done based on the quasi log transformed data and not only age but several other explanatory variables (Table 1).

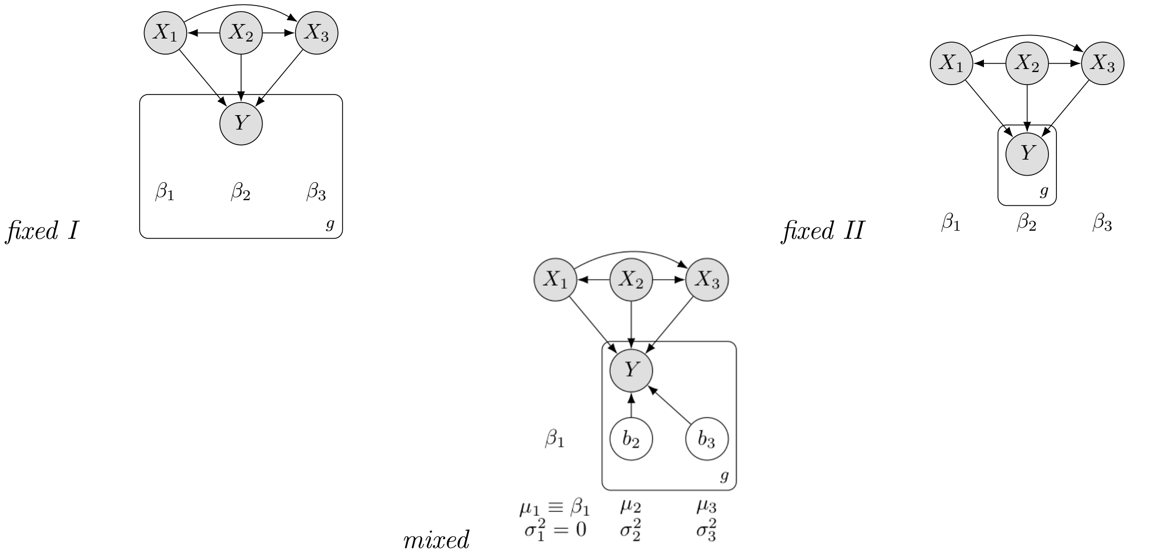

Supplementary Figure 5: Three model structures: two *fixed* (upper left and right) and a *mixed* (lower middle) effects multiple regression model. In all three model structures the read count ratio Y_g—for several genes g—depends somehow on three explanatory variables X_j like Age or PMI (Table 1). For each gene g the probabilistic dependence is mediated by fixed $\beta_{1g}, \beta_{2g}, \beta_{3g}$ or random b_{2g}, b_{3g} regression coefficients. *fixed II* is a constrained version of the *fixed I* model structure such that $\beta_{jg_1} = \beta_{jg_2} = ... \equiv \beta_j$, which means that the effect of X_j on Y does not vary across genes in *fixed II*. The *mixed* model differs from *fixed I* in the way coefficients across genes vary for a given explanatory variable X_j. In the *fixed I* model structure there is no connection among $\beta_{jg_1}, \beta_{jg_2}, ...$, which means that the way Y_g, the read count ratio for gene g depends on variable X_j is completely separate from how the read count ratio for any other gene g' (i.e. $Y_{g'}$) depends on X_j. Consequently, the gene-specific substructures of *fixed I* contain no information on each other. This limitation is overcome with the *mixed* model structure because a set of coefficients across genes—e.g. the set $\{b_{2g}\}_g$)—is modeled as a random sample from a normal distribution with parameters μ_2 and some $\sigma_2^2 > 0$. Thus μ_2 and σ_2^2 constitute information on the effect that is shared across all genes so that genes "borrow strength from each other". When $\sigma_j^2 = 0$ in the *mixed* model then all parameters $\{b_{jg}\}_g$ for X_j are fixed at $\mu_j \equiv \beta_j$, which is characteristic to the *fixed II* model structure. In the *mixed* model structure this is seen for X_1, which therefore has the same effect on Y_g for every gene g. In this example all explanatory variables are continuous in both models. Any categorical explanatory variable (factor) X_j with K levels would lead to $K - 1$ fixed or random coefficients $\beta_{j_1 g}, ..., \beta_{j_{K-1} g}$ or $b_{j_1 g}, ..., b_{j_{K-1} g}$ for any gene g, respectively. Moreover, if the effect of that categorical X_j is random then it is possible to have a continuous $X_{j'}$ with a random intercept and slope with respect to X_j. In fact the *mixed* model structure (lower middle) is equivalent to another one (not shown), where "Gene" is a random factor X_{Gene} with random slope for the effects of X_2 and X_3.

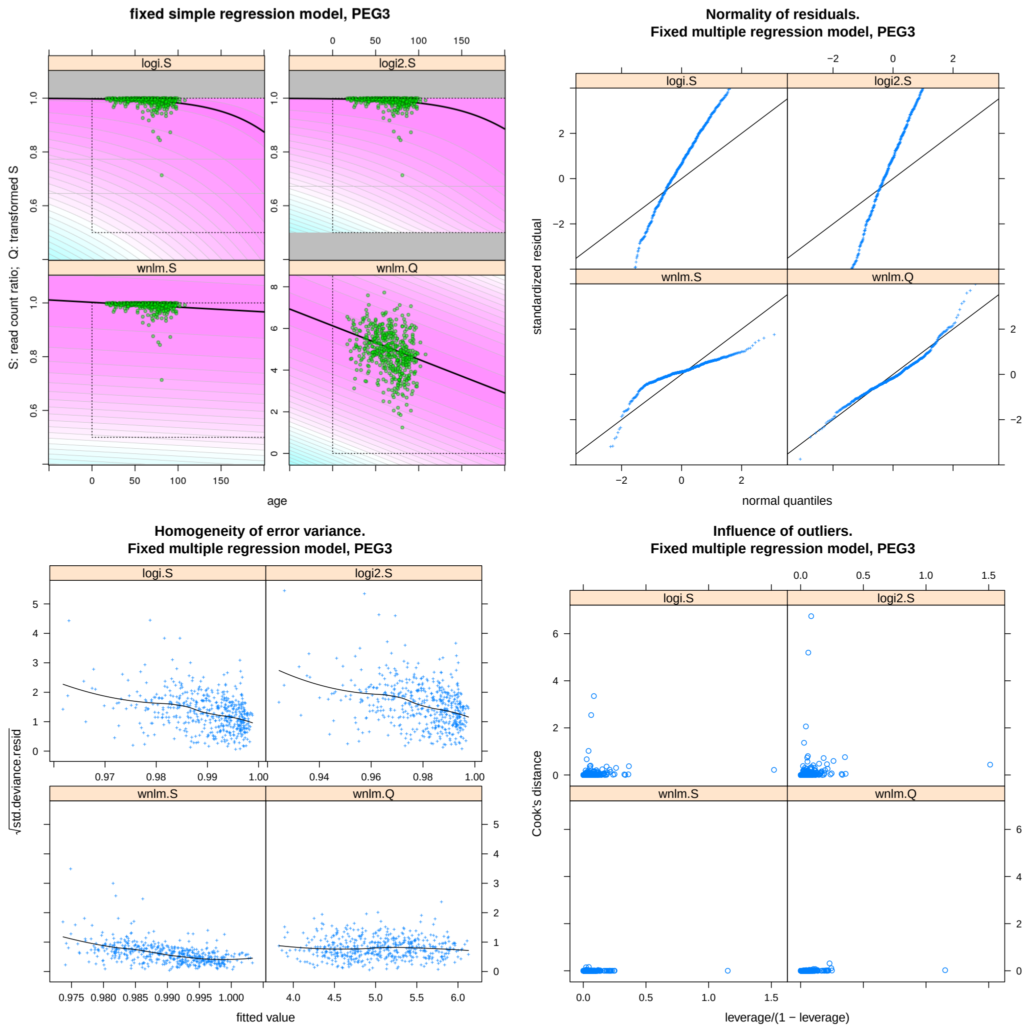

Supplementary Figure 6: Fitting various fixed regression models, named logi.S, logi2.S, wnlm.S, wnlm.Q (Table 2), on the read count ratio data for the PEG3 gene. Results for models unlm.S, unlm.Q, unlm.R, wnlm.R are omitted for clarity and redundancy. In particular, unlm.Q gave as good fit as wnlm.Q. *Upper left:* Fitted curves (black lines) and sampling probabilities (magenta-white-cyan color gradient) of a version of the four models that is simple in the sense that Age is the only explanatory variable. Simple regression is used for this illustration only. For inference and all other plots in this figure multiple regression was performed, where Age is only one of several explanatory variables (Table 1). *Upper right (Normality of residuals):* analysis of the normality of the standardized residuals of fits suggests wnlm.Q is the best fitting model. *Lower left (Homogeneity of error variance):* Similar conclusion can be made by inspecting how the standardized deviance residuals depends on the fitted value. Goodness of fit is indicated by the lack of such dependence. Black curve: LOESS data smoother. *Lower right (Influence of outliers):* Influence of each individual on the fit quantified by Cook's distance (y-axis). This is plotted against a function of leverage, which quantifies a subcomponent of influence that is restricted to explanatory variables (i.e. individuals with extreme age, PMI,...). In ideal case all data points are expected to influence the fit to the same degree and thus have short Cook's distance.

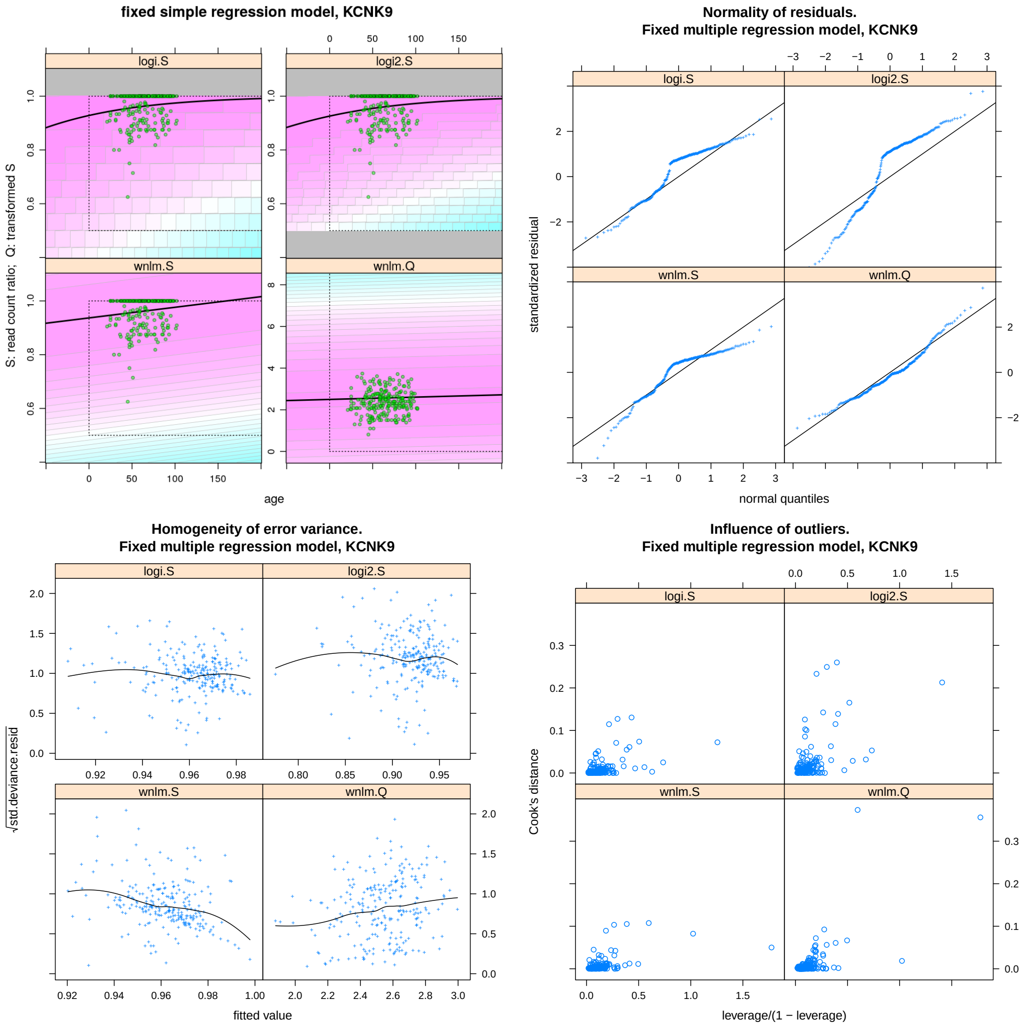

Supplementary Figure 7: Fitting various fixed regression models on read count ratio data for the KCNK9 gene. See the legend of Fig. 6 for further details.

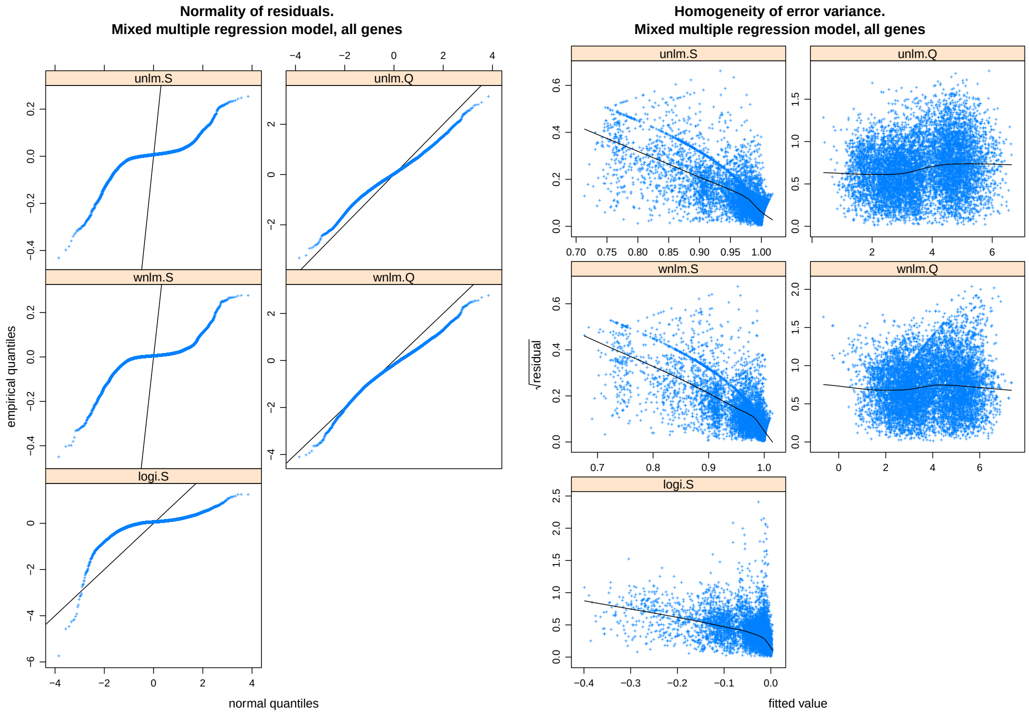

Supplementary Figure 8: Fitting various mixed regression models, named logi.S, logi2.S, wnlm.S, wnlm.Q (Table 2), on the read count ratio data for all imprinted genes jointly. Results for models unlm.S, unlm.Q, unlm.R, wnlm.R are omitted for clarity. The plots suggest that unlm.Q and wnlm.Q fit the data the best. See the legend of Fig. 6 for further details. For its faster convergence (not shown) unlm.Q was selected as the favored model for statistical inference.

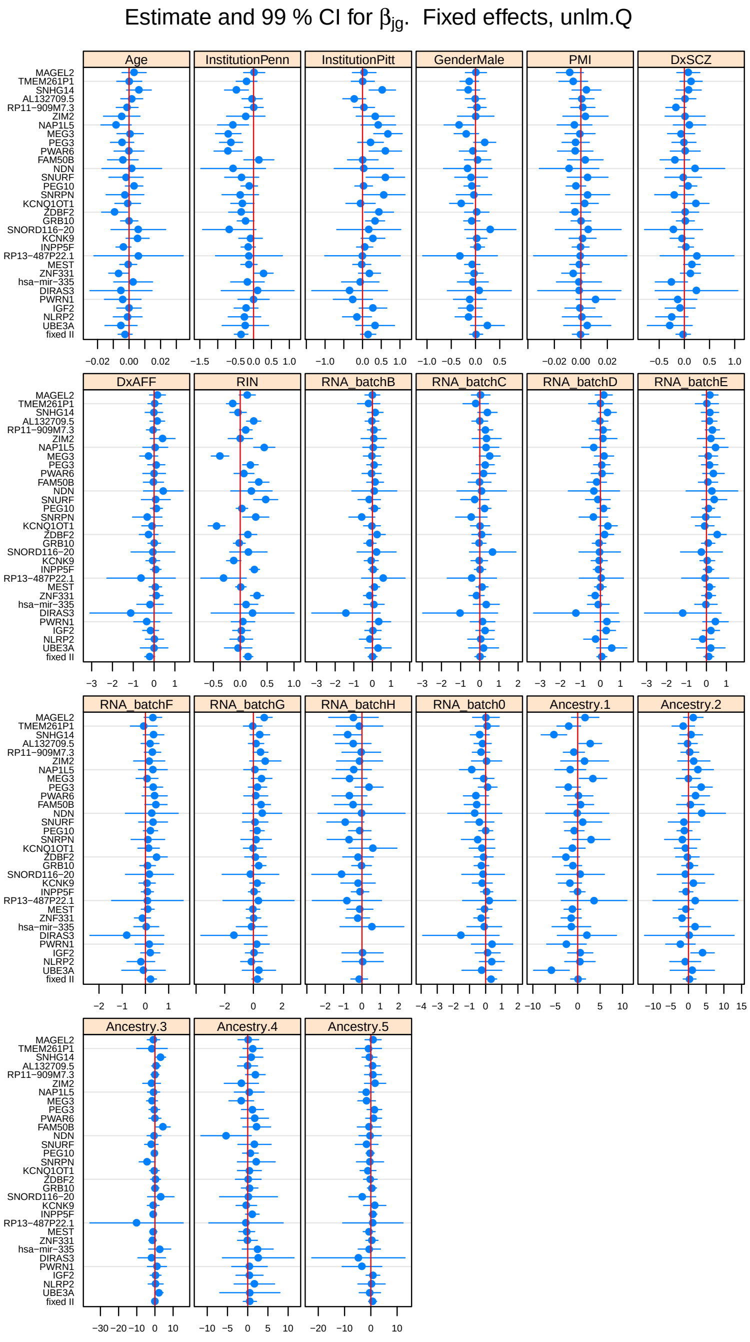

Supplementary Figure 9: Estimated coefficients β_{jg} and 99% confidence intervals for gene g (y-axis) and fixed effect j (panel headers) under the *fixed I* model structure (Fig. 5). Below gene UBE3A the label fixed II indicates the gene-independent estimate under the *fixed II* model (Fig. 5). Positive and negative coefficient indicates direct positive and negative dependence of the given gene's read count ratio on age, respectively. Compare with Fig. 5 and 10.

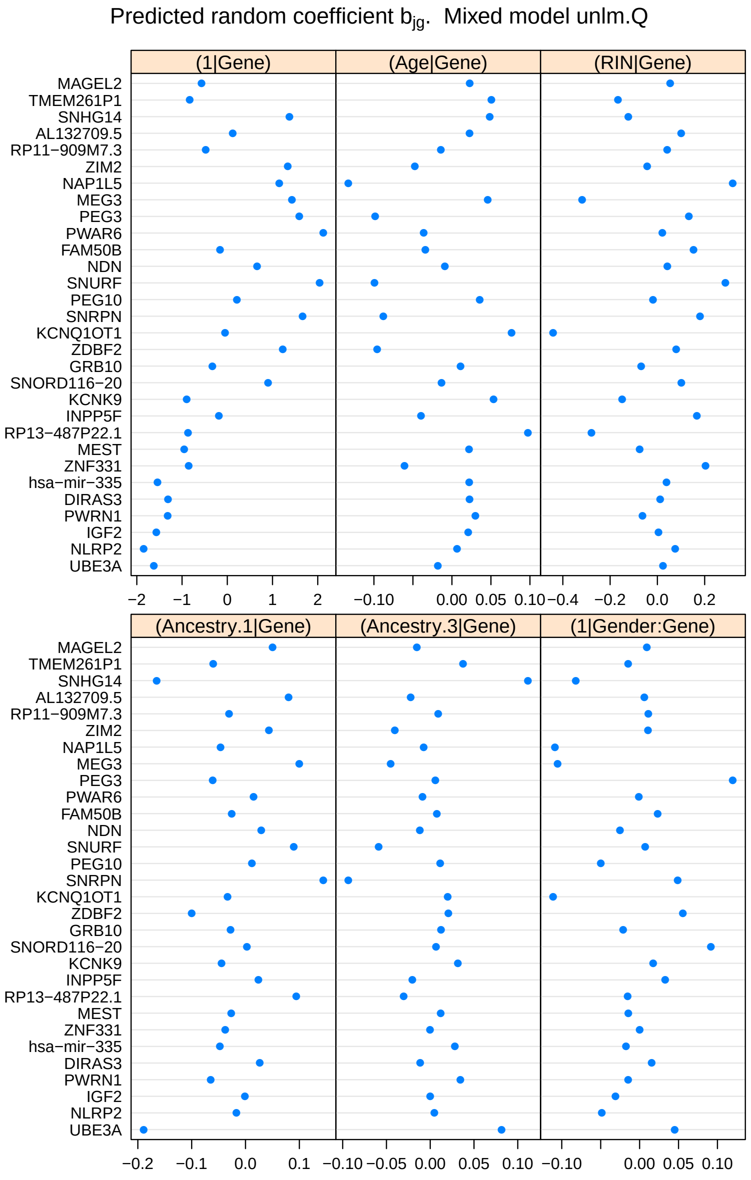

Supplementary Figure 10: Predicted random coefficients b_{gj} for gene g (y-axis) and random effect j (panel headers) under the *mixed* model structure (Fig. 5). Positive and negative coefficient indicates direct positive and negative dependence of the given gene's read count ratio on age, respectively, while zero coefficient suggests independence of age. Compare with Fig. 5 and 9.

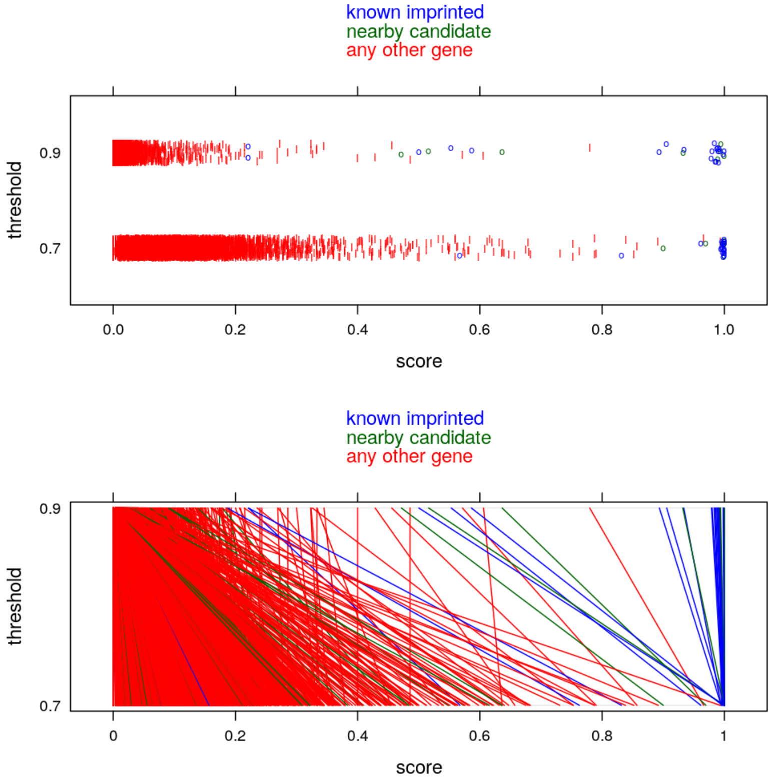

Supplementary Figure 11: *Top:* Distribution of gene score as defined as $1 - \text{ECDF}(0.9)$ (threshold 0.9) or as $1 - \text{ECDF}(0.7)$ (threshold 0.7). *Bottom:* The same gene scores are shown as in the top graph with the additional information that points corresponing to the same genes are connected by straight lines. This demonstrates that gene rank is roughly consistent between the two thresholds.

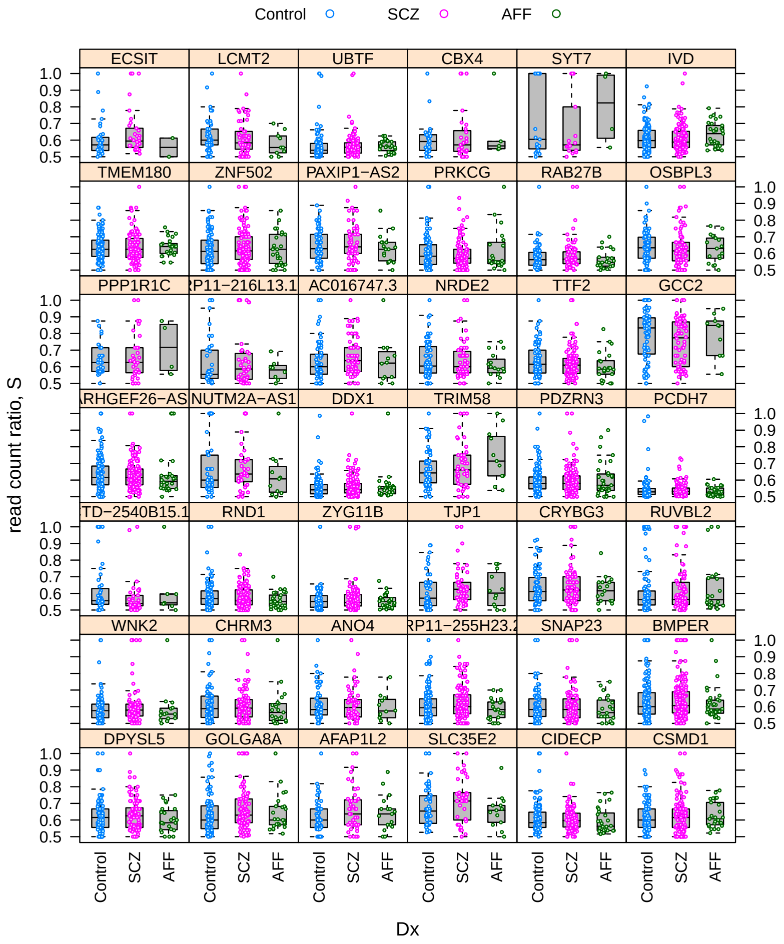

Supplementary Figure 12: Distribution of read count ratio in Control, Schizophrenic (SCZ) and Affective spectrum disorder (AFF) individuals for randomly selected not imprinted genes.

2 Supplementary Tables

explanatory variable	levels
Age	
Institution	[MSSM], Penn, Pitt
Gender	[Female], Male
PMI	
Dx	[Control], SCZ, AFF
RIN	
RNA_batch	[A], B, C, D, E, F, G, H, 0
Ancestry.1	
⋮	
Ancestry.5	

Supplementary Table 1: *Left column:* explanatory variables of read count ratio. *Right column:* levels of each factor-valued (i.e. categorical) variable. Square brackets [...] surround the baseline level against which other levels are contrasted. *Abbreviations:* PMI: post-mortem interval; Dx: disease status; AFF: affective spectrum disorder; SCZ: schizophrenia; RIN: RNA integrity number; Ancestry.k: the k-th eigenvalue from the decomposition of genotypes indicating population structure.

model family	abbrev.	response var.
*u*nweighted *n*ormal *l*inear	unlm	S, Q, or R
*w*eighted *n*ormal *l*inear	wnlm	S, Q, or R
*logi*stic	logi	S
*logi*stic, $\frac{1}{2}\times$ down-scaled link fun.	logi2	S

Supplementary Table 2: Fitted regression model families, in which the response variable is the read count ratio with or without some transformation: S—untransformed, Q—*q*uasi-log-transformed, and R—*r*ank-transformed read count ratio. Diagnostic plots (Fig. 8) and monitoring convergence suggested that the unlm.Q combination allows the best fit for several linear predictors tested.

data subset / predictor term	odd ranked genes ΔAIC	odd ranked genes p-value	even ranked genes ΔAIC	even ranked genes p-value
(1 | Gene)	-61.2	5.7×10^{-14}	-59.2	1.5×10^{-13}
(1 | Dx)	2.0	1.0	2.0	1.0
(1 | Dx : Gene)	1.9	0.71	0.0	0.16
Age	0.0	0.16	2.0	0.86
(Age | Gene)	-11.8	5.8×10^{-4}	5.1	0.43
Ancestry.1	-0.4	0.12	1.8	0.66
(Ancestry.1 | Gene)	-40.1	1.3×10^{-9}	-18.5	2.9×10^{-5}
Ancestry.3	1.7	0.59	1.6	0.54
(Ancestry.3 | Gene)	-13.3	2.9×10^{-4}	6.0	0.55
(1 | Gender)	2.0	1.0	0.7	0.25
(1 | Gender : Gene)	-2.2	4.0×10^{-2}	0.1	0.17

Supplementary Table 3: Results based on mixed models fitted on two subsets of the data: the first subset corresponds to odd ranked genes, while the second to even ranked genes (see odd and columns in Fig. 4, Fig. 5, and Fig. 4). A few findings are notable. First, these results are less significant in general than those obtained from the full data set (Table 1), which follows from the reduction both in the number of data points and in the number of genes. Second, the term (Age | Gene) is significant for odd ranked genes but not for even ranked genes. This agrees with the qualitative pattern seen in Fig. 4, where the genes in the odd columns show a pronounced variability with respect to age dependence but genes in even columns do not. The differences between the two subsets are also explained in part by the fact that there happen to be more missing data for even ranked genes.
